# Effects of α-pinene on the pinewood nematode (*Bursaphelenchus xylophilus*) and its symbiotic bacteria

**DOI:** 10.1371/journal.pone.0221099

**Published:** 2019-08-19

**Authors:** Xu Wang, Yanxue Yu, Jianjun Ge, Bingyan Xie, Shuifang Zhu, Xinyue Cheng

**Affiliations:** 1 College of Life Sciences, Beijing Normal University, Beijing, China; 2 Chinese Academy of Inspection and Quarantine, Beijing, China; 3 Institute of Vegetables and Flowers, Chinese Academy of Agricultural Sciences, Beijing, China; University of Illinois at Urbana-Champaign, UNITED STATES

## Abstract

The pinewood nematode (PWN), *Bursaphelenchus xylophilus*, is an important plant-parasitic nematode that can cause severe mortality of pine trees. This PWN-induced harm to plants may be closely related to the abundance and diversity of the symbiotic microorganisms of the parasitic nematode. In this study, nematodes were divided into untreated and antibiotic-treated groups. Nematodes were treated by fumigation with different amounts of α-pinene, and the resultant mortality rates were analyzed statistically. Concentrations of symbiotic bacteria were calculated as colony-forming units per nematode. High-throughput sequencing was used to investigate the bacterial community structure. The results showed that the mortality of nematodes increased slightly with an increasing concentration of α-pinene, and nematodes untreated with antibiotics were more sensitive to α-pinene than those treated with antibiotics. The highest abundance of symbiotic bacteria was obtained via medium and low levels of α-pinene, but for which community diversity was the lowest (Shannon and Simpson indexes). The proportion of *Pseudomonas* spp. in the symbiotic bacteria of nematodes without antibiotics was relatively high (more than 70%), while that of *Stenotrophomonas* spp. was low (6%–20%). However, the proportion of *Stenotrophomonas* spp. was larger than that of *Pseudomonas* spp in the symbiotic bacteria associated with the antibiotic-treated nematodes. *Pseudomonas* sp. increased after pinene treatment, whereas *Stenotrophomonas* spp. decreased. These results indicate that although α-pinene has low toxicity to PWNs over a short time period, α-pinene ultimately influences the abundance and community diversity of the symbiotic bacteria of these nematodes; this influence may potentially disturb the development and reproduction of nematodes in the process of infecting pine trees.

## Introduction

The pinewood nematode (PWN), *Bursaphelenchus xylophilus*, causes pine wilt disease (PWD). Pine species (*Pinus massoniana*) produce many secondary metabolites to defend against pests. Among them, terpenoids are one of the most important components. Specifically, α-pinene comprises a large proportion of terpenoids in pine trees [1–3]. After the PWN infects the pine, the contents of terpenoids in the parenchyma cells and trachea of the host PWN increase [4–8]. These compounds have nematicidal properties and inhibit nematode-propagation activities at a certain concentration in PWNs [9–11]. The recombinant venom allergen protein (VAP), which is encoded by the toxic allergen-like protein gene (*Bx-vap-1*), is specifically expressed in esophageal-gland cells of PWNs and has been used to mimic the oral secretions of PWNs. It has been shown that inoculating recombinant VAP into pine trees up-regulates the expression of the α-pinene synthase gene and increases the content of α-pinene [12]. Therefore, the ability of PWN to metabolize and detoxify these compounds is important. However, as previously reported, cytochrome-P450 enzymes are the main enzymes that metabolize xenobiotics in PWN, similar to that observed in *Caenorhabditis elegans*. A few specific enzymes have been shown to be involved in the limonene- and pinene-degradation pathways in the genomae of PWN. [13, 14]. Previous studies in our laboratory have shown that, the genomes of PWN symbiotic bacteria are rich in genes in the limonene and pinene degradation pathways. Moreover, symbiotic bacteria may play a key role in the response of PWNs to host-defensive compounds, especially α-pinene [15]. Therefore, it is necessary to investigate the effects of α-pinene on PWNs and their symbiotic bacteria.

In the present study, PWNs were treated with α-pinene via fumigation. The effects of α-pinene on PWNs were analyzed by quantifying the mortality rate of nematodes. Counting colony-forming units (CFUs) per nematode and high-throughput sequencing were used to analyze the quantity and community diversity of PWN symbiotic bacteria. The results suggest that α-pinene induces little to no toxicity in PWNs, but significantly affect the quantity and community structure of their symbiotic bacteria.

## Materials and methods

### Sample collection

One *B*. *xylophilus* strain (ZJSS) was selected from a culture in our library. This nematode originated from chips of a pine tree that suffered from PWD infection in Zhejiang Province of China. The Baermann funnel technique was employed for nematode isolation [16].

### Nematode pretreatment

ZJSS was divided into the antibiotic-untreated PWN group and the antibiotic-treated PWN group. The process of antibiotic treatment consisted of sterilizing nematodes with 3% H_2_O_2_ for 10 min, washing them three times with sterile water, immersing them in 1% thimerosal for 30 min, and then washing them three times with sterile water. Nematodes were then transferred to 0.5% of an antibiotic mixture (streptomycin sulfate, ampicillin, gentamicin, and cephalosporin) overnight and were washed three times with sterile water.

Two groups were cultured on fungal mats of *Botrytis cinerea* and were grown on potato-dextrose agar (PDA) plates at 25 ± 1°C in darkness for approximately 10 days. The cultured nematodes were separated from the culture medium with distilled water in the biological safety cabinet (BSC). The standard nematode suspensions were configured with sterilized water for testing.

### Fumigation of nematodes by α-pinene

A standard nematode suspension was prepared by an appropriate dilution with distilled water (approximately 5,000 nematodes mL^−1^), and the number of nematodes was counted under a binocular microscope (20×) in the BSC. We added 1 mL of nematode suspension onto the 2% water agar. Meanwhile, 4 mL (L-level), 8 mL (M-level), and 16 mL (H-level) of (-)- α-pinene (98% purity, Energy Chemical, China) were separately injected into a cotton ball, and then the cotton balls were placed in the center of the dish cover as treatments. A control consisted of a cotton ball injected with sterile distilled water. Each treatment had five replicates. To ensure fumigation, the culture dish was placed upside down in order for the cotton ball to make contact underneath the nematode. Treated and control nematodes were kept at 25 ± 1°C in darkness for 48 h (S3 Fig).

### Nematode mortality rate

All of the nematodes were immersed in distilled water for one hour in the BSC after treatment. A binocular microscope was used to confirm that no residual nematodes were left in the plate. Next, samples were washed three times with sterile water. The nematode suspensions were transferred to a 1.5-mL centrifuge tube, and were then diluted to 1 mL with sterile water. The surviving nematodes were counted. Nematodes were defined as dead if their bodies were straight and they did not move, even after touching them with a needle for 5 sec. The mortality ratio (MR) was determined using the formula: MR = (T_a_ − T_b_)/T_a_ × 100, where T_a_ is the number of nematodes before treatment and T_b_ is the number of living nematodes after treatment. SPSS statistics 22 was used for analysis of differences in nematode mortality between the treatment and control groups.

### Bacteria concentration

One-hundred μL of the treated 1-mL nematode suspension was randomly extracted into a 1.5-mL centrifuge tube. The centrifuge tube was placed in liquid nitrogen for 8 sec and the contents were then grinded with an electric grinder. The slurry was diluted using sterile water and was plated on nutrient agar (NA). Samples were then cultured at 30 ± 1°C in the darkness for 24 h. The number of colonies was counted and the CFU of each nematode was calculated. All of the treatments were compared by analysis of variance (ANOVA) using SPSS.

### Bacterial DNA extraction, PCR, and sequencing

Another 100 μL of nematode suspension was extracted and grinded via the same procedure described above. Total bacteria DNA was extracted using a TIANamp Bacteria DNA Kit (TIANGEN, China). Bacterial 16S rRNA genes were PCR amplified using a set of two broadly conserved, degenerate primers targeting the V3–V4 variable regions of the 16S gene (341F: 5′–CCT ACG GGN GGC WGC AG–3′; 805R: 5′–GAC TAC HVG GGT ATC TAA TCC–3′).

The PCR reaction was conducted using a thermal instrument (Applied Biosystems 9700, USA) to subject the samples to the following conditions: 95°C for 3 min (initial dissociation); first five cycles of 95°C for 30 s (denaturation); 45°C for 30 s (annealing); 72°C for 30 s (extension), followed by 20 cycles of 95°C for 30  s (denaturation); 55°C for 30  s (annealing); 72°C for 30 s (extension) and a final extension at 72°C for 5 min. Negative non-template controls were run for each barcode-primer pair to test for reagent contamination. Each sample was amplified and then pooled together for analysis. The PCR products were analyzed by gel electrophoresis and were purified using a PCR Purification Kit (Sangon, Shanghai, China). Sequencing was performed on an Illumina MiSeq 2 × 250 platform (Illumina Miseq, USA). A total of 40 samples within eight treatments were sequenced.

### Sequence quality control and processing

The QIIME v.1.8.0 [17] pipeline was mostly used for data-quality controls and analyses. Raw-sequence reads were filtered to meet the minimum and maximum lengths of 200 bp and 450 bp with no ambiguous base calls. Sequences that contained more than one ambiguous base call (N) that did not also have a complete barcode and primer at one end were eliminated. A read was discarded if it was identified as a putative chimera by UCHIME [18]. The sequences that passed the above procedure were denoised to correct for potential sequencing errors at the 99% level using UCLUST [18]. Operational Taxonomic Units (OTUs) were defined using UCLUST v1.1.579, which has been confirmed to generate satisfactory and comparable numbers of OTUs [19]. A threshold of ≥ 97% was used for OTU identity at the genus level. The RDP classifier (version 2.10) software was used [12] to classify the sequences according to the taxonomy proposed by Garrity et al. [20].

### Bacterial-community diversity analysis

The Chao, ACE, Shannon diversity index and Simpson diversity index were calculated based on the sample OTU data during alpha diversity analysis conducted using MOTHUR [21]. The Mann–Whitney U test was used to determine significance. The species-classification bar (top-10 genera) and species-abundance heat map for each group of samples were then generated. Principal component analysis (PCA) was applied to the rarefied OTU data to reflect the main characteristics of the data. This analysis was computed using R Software. The metagenome functional content from 16S rRNA was predicted using PICRUSt (Phylogenetic Investigation of Communities by Reconstruction of Unobserved States) [22]. Based on the third-level KEGG pathways results of the PICRUSt function, the significance of the difference in abundances among groups was compared.

## Results

### PWN mortality rate

**Fig 1:**
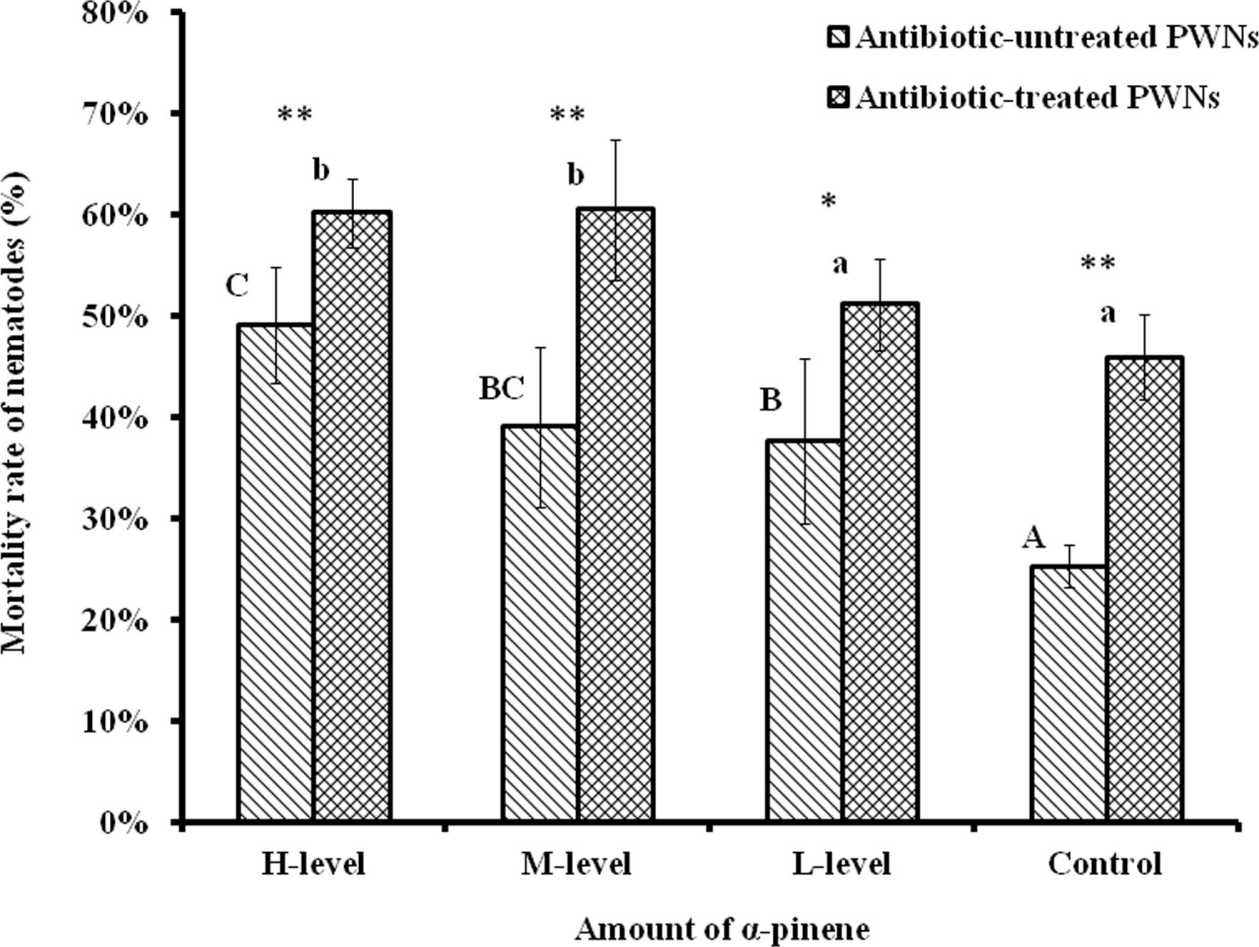
Mortality rate of nematodes. Different letters indicate significant (*p* < 0.05) differences in nematode mortality in corresponding nematode groups, as determined by Tukey's HSD test. Dots represent differences between two nematode groups (*p < 0.05, ** *p* < 0.01).

All of the treatments showed that the mortality rates of the two PWN groups increased as the α-pinene amounts increased (S1 Table), compared with that of control PWNs. The mortality rate of the antibiotic-treated PWNs was significantly higher than that of the antibiotic-untreated PWNs, regardless of whether the samples were treated with α-pinene (Fig 1). There was no significant correlation between antibiotic treatment and different amounts of α-pinene treatment (*p* = 0.133; S2 Table). For the antibiotic-untreated PWNs, the mortality rates of nematodes were 37.65%, 39.03%, and 49.10% at an L-level, M-level and H-level of α-pinene, respectively. With the increase of pinene amount, the mortality rate increased significantly. For the antibiotic-treated PWNs, the mortality rate of nematodes treated with an L-level (51.14%) was slightly higher than that of the control (45.94%). The mortality rates of nematodes treated with M-level (60.48%) and H-level pinene (60.19%) were significantly higher than that of the control. (Fig 1). These results showed that antibiotic-untreated PWNs were more sensitive to α-pinene than antibiotic-treated PWNs.

### Bacteria CFUs

**Fig 2:**
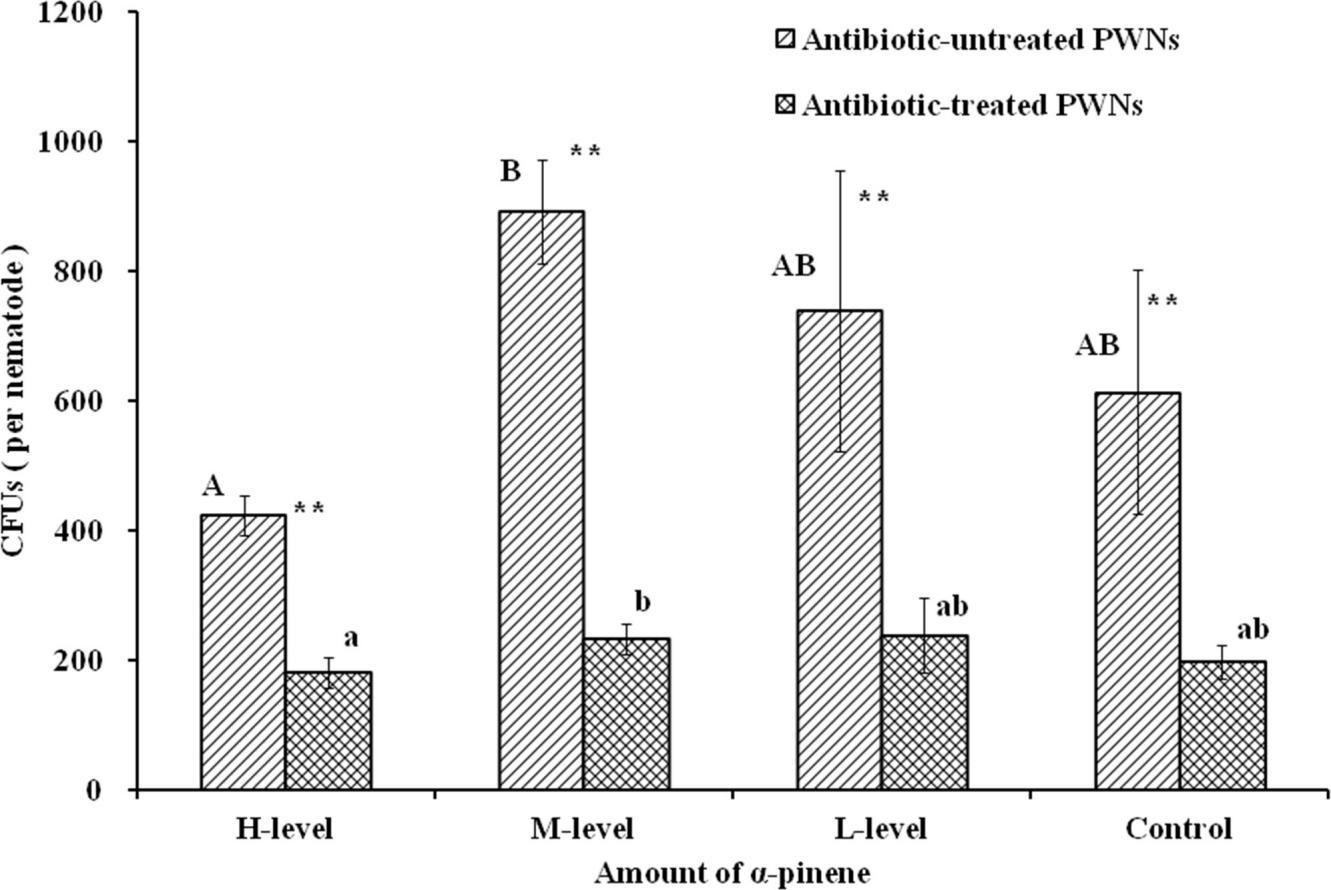
CFUs of bacteria. Different letters indicate significant (*p* < 0.05) differences in CFUs of corresponding nematode groups, as detected by Tukey's HSD test. Dots represent differences between two nematode groups (*p < 0.05, ** *p* < 0.01).

The CFUs of symbiotic bacteria of the antibiotic-treated PWNs were lower than those of the antibiotic-untreated PWNs at all of the pinene treatment levels (Fig 2). For the antibiotic-untreated PWNs, the CFUs of the L-level (738 per nematode) and M-level (891 per nematode) increased with the increasing amount of pineneand the lowest CFUs of bacteria (422 per nematode) were obtained for the H-level of pinene (S1 Table). The range of change of CFUs of symbiotic bacteria in the antibiotic-treated PWNs was lower than that in the antibiotic-untreated PWNs. The CFUs at the L-level and M-level were slightly higher than those of the control. Similar to the antibiotic-untreated PWNs, the lowest CFUs were detected for the H-level of pinene.

### Species-abundance analysis of symbiotic bacteria

**Fig 3:**
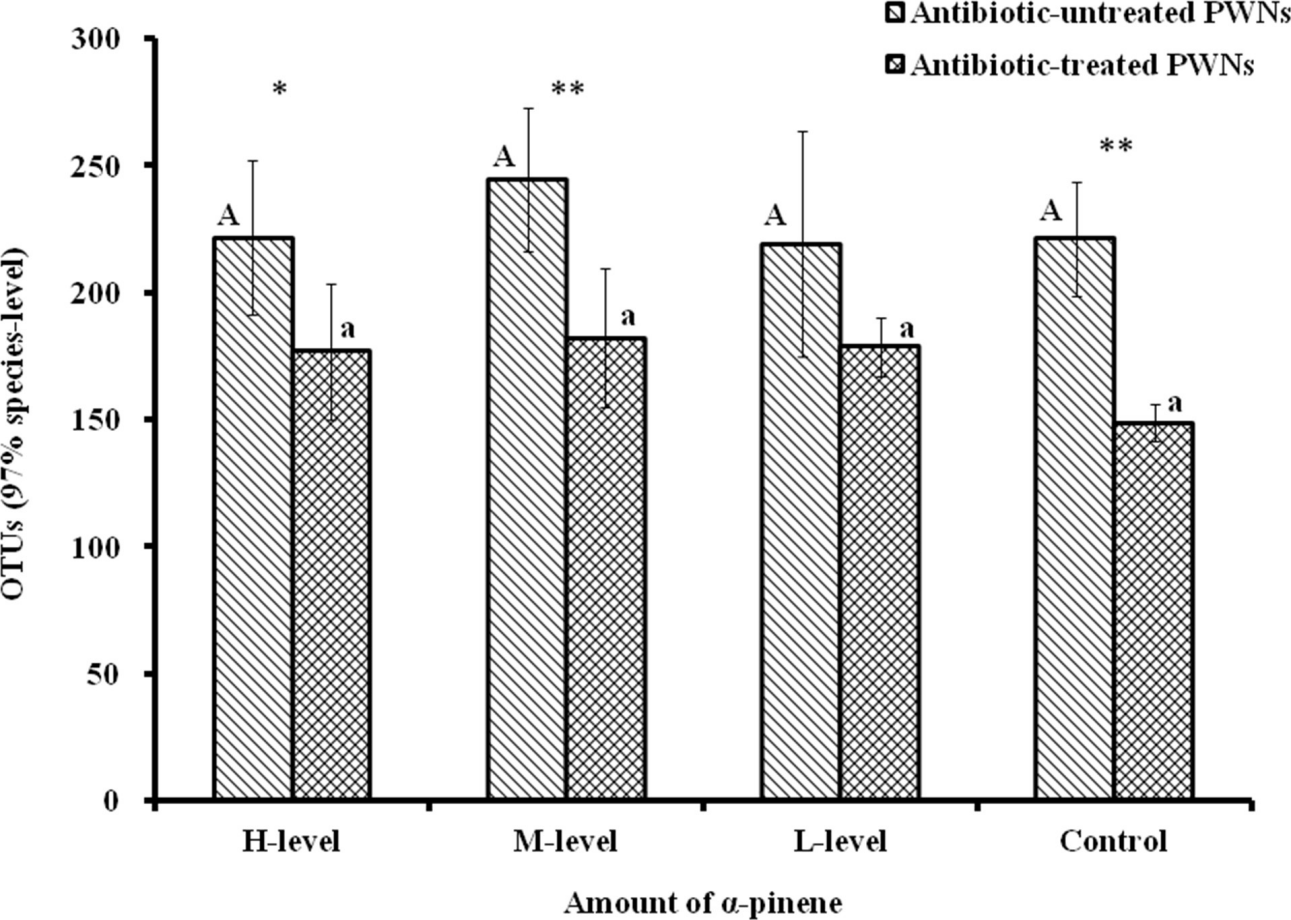
OTUs of bacteria. Different letters indicate significant (*p* < 0.05) differences in OTUs of corresponding nematode groups, as detected by Tukey's HSD test. Dots represent differences between two nematode groups (*p < 0.05, ** *p* < 0.01).

Next, 16S rRNA sequences of the PWN symbiotic bacteria were filtered using the QIIME software. Overall, 2,062,368 high-quality sequences were obtained from the 40 samples and about 51,559 sequences per sample were identified and assigned OTUs (S3 Table). The average length of the assembled reads was 429 bp. The number of OTUs of bacteria associated with the antibiotic-untreated PWNs was greater than that of the antibiotic-treated PWNs (S4 Table). The smallest number of OTUs was detected in the control group of the antibiotic-treated PWNs (149±7.463), while the antibiotic-untreated PWNs treated with the M-level had the most OTUs (244±28.112; Fig 3). We upload these data to Figshare (https://doi.org/10.6084/m9.figshare.8117867.v1), and users can find relevant data by looking up relevant folders after registration for the service.

**Fig 4:**
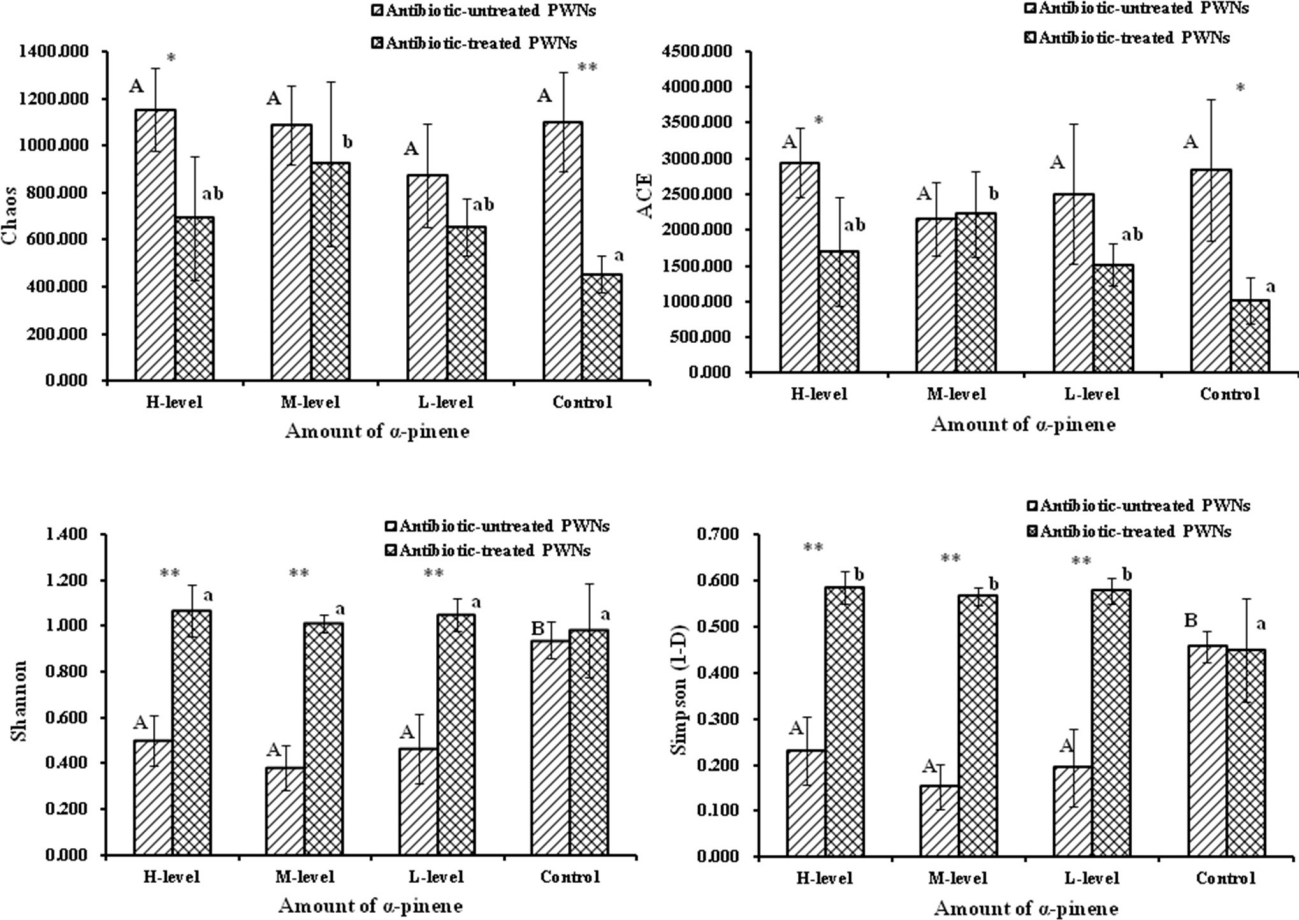
Alpha diversity index of bacteria community. Different letters indicate significant (*p* < 0.05) differences in OTUs of corresponding nematode groups, as detected by Tukey's HSD test. Dots represent differences between two nematode groups (*p < 0.05, ** *p* < 0.01).

The coverage estimations of total samples were 100% of symbiotic bacteria of PWNs. Four alpha-diversity-analysis indices were calculated, including Chao, ACE, the Shannon index, and the Simpson index (Fig 4). The Chao and ACE results showed that the richness of the community of bacteria associated with the antibiotic-untreated PWNs were higher than those of the antibiotic-treated PWNs, especially in the H-level pinene treatments and the control, except for the ACE in the M-level pinene treatments. The Chao and ACE indices of bacteria varied greatly among different treatments of the antibiotic-treated PWNs, and the highest indices of Chao and ACE were obtained for the M-level α-pinene treatment. However there was no significant differences in the Chao and ACE among several treatments of the antibiotic-untreated PWNs. The variations of bacterial diversity among several treatments in the antibiotic-untreated PWNs were more obvious than those in the antibiotic-treated PWNs. The Shannon and Simpson indices of bacteria of antibiotic-untreated PWNs decreased after α-pinene treatment. The lowest community diversity was detected for the M-level treatment. However, there was no significant difference in diversity among different treatments of the antibiotic-treated PWNs. The Shannon index and the Simpson index indicated that the community diversity of bacteria associated with the antibiotic-treated PWNs after treatment with pinene was significantly higher than that of the antibiotic-untreated PWNs (Fig 4).

### Community-structure analysis of symbiotic bacteria

**Fig 5:**
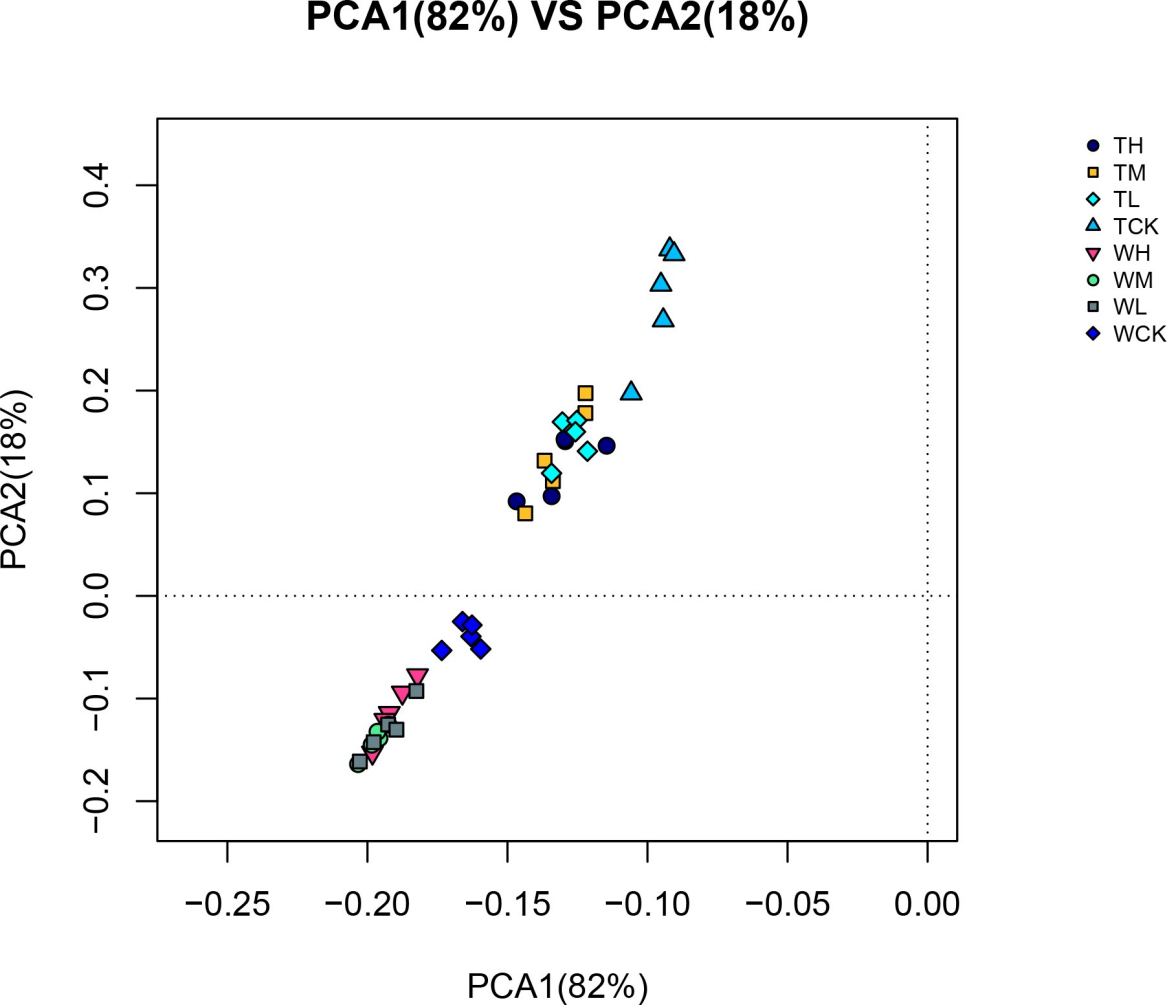
PCA of OTUs. WH, WM, WL, and WCK denote the antibiotic-untreated PWNs receiving the three different levels of α-pinene, as well as the control-group (WCK). TH, TM, TL, and TCK denote the antibiotic-treated PWNs receiving the three different levels of α-pinene, as well as the control-group (TCK).

The Bayesian algorithm was used for taxonomic analysis of OTUs differentiated based on a cutoff of 97% similarity. The results showed that the dominant phylum of symbiotic bacteria was Proteobacteria (S1 Fig), and that the reads of Proteobacteria accounted for more than 98% of the total reads in each sample. Gammaproteobacteria was the most dominant class, with its reads representing 87% of the total reads (S2 Fig). At least 80% of the OTUs were Proteobacteria, while Gammaproteobacteria accounted for at least 55%. The differences in phylum level between samples were not significant, but PCA analysis (Fig 5) indicated that there was a separation between the two groups.

**Fig 6:**
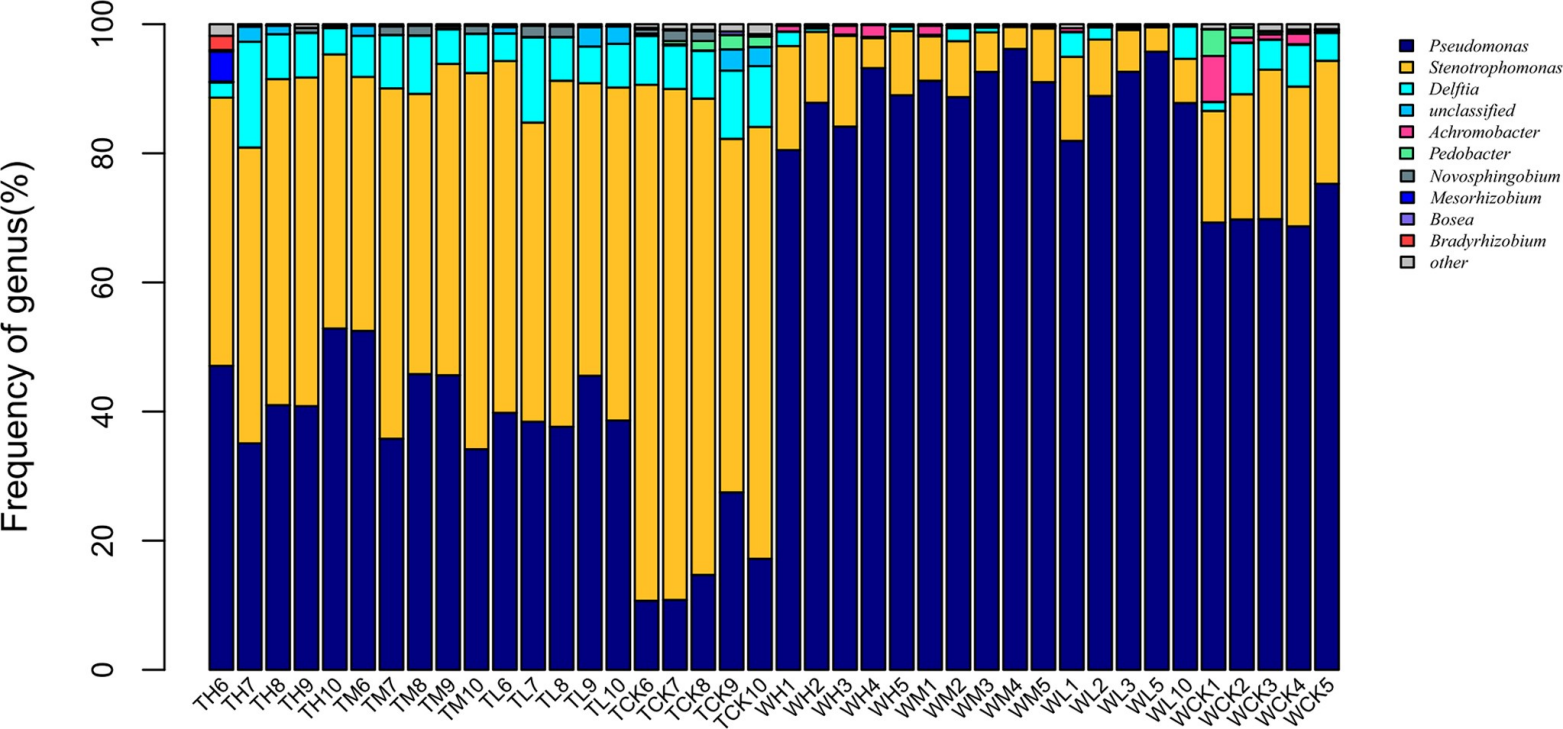
Bacterial composition at the genus level. WH1-5, WM1-5, WL1-3,5,10, and WCK1-5 denote the antibiotic-untreated PWNs receiving the three different levels of α-pinene, as well as the control group (WCK1-5). TH6-10, TM6-10, TL6-10, and TCK6-10 denote the antibiotic-treated PWNs receiving the three different levels of α-pinene, as well as the control group (TCK6-10).

The bar graphs of the top-10 genera showed that *Pseudomonas* spp. and *Stenotrophomonas* spp. were dominant (Fig 6), followed by *Delftia* spp., Sphingomonadaceae unclassified and *Achromobacter* spp. The sum of these species reads represented more than 97% of the total number in each sample. There were significant differences in the proportions of the two dominant genera of the two groups. Specifically, the proportion of *Pseudomonas* spp. in the antibiotic-untreated PWNs was high (more than 70%), while that of *Stenotrophomonas* spp. was relatively low (6%–20%). Following pinene treatment, the ratio of *Pseudomonas* spp. increased from 70% to 91%, while the proportion of *Stenotrophomonas* spp. decreased from 20% to 6%. There were no significant differences among the three levels of pinene treatment; however, the proportion of *Stenotrophomonas* spp. in the antibiotic-treated PWNs was larger than that of the *Pseudomonas* spp. Following pinene treatment, the proportion of *Stenotrophomonas* spp. decreased from 70% to 50%, whereas the ratio of *Pseudomonas* spp. increased from 16% to 40%. Under the treatment of pinene, the dominant population abundances of the two groups changed significantly, and the changes were roughly the same. *Pseudomonas* sp. increased under treatment with pinene, while *Stenotrophomonas* spp. and *Delftia* spp. decreased in response to pinene treatment.

### Functional-prediction analysis of symbiotic bacteria

**Fig 7:**
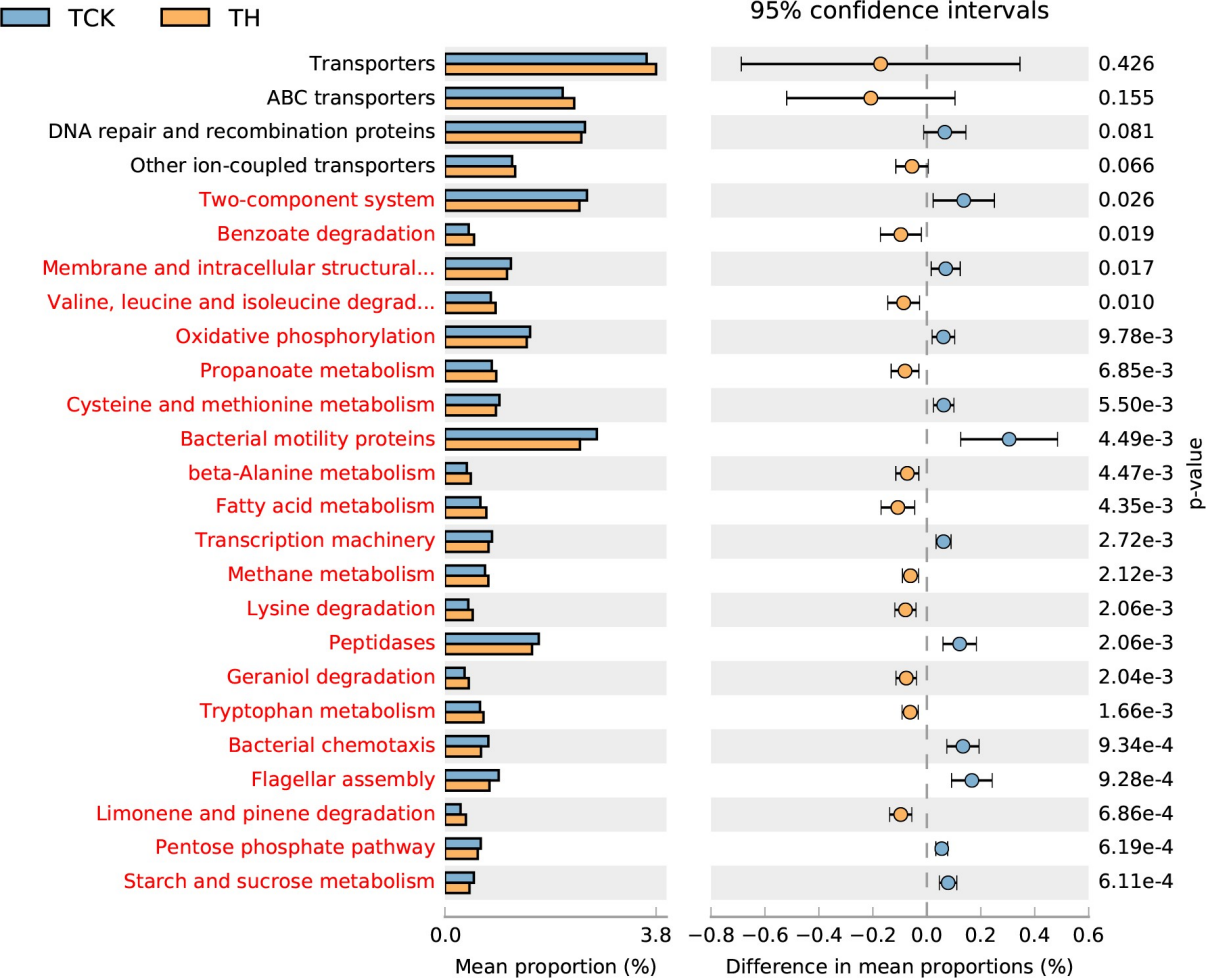
Top-25 functional abundance-difference analysis between the TCK and TH. TH denotes the antibiotic-treated PWNs receiving the H-level of α-pinene. TCK denotes the antibiotic-treated PWNs in the control group. The left side of the chart shows the abundance of different functional groups in the two groups of samples. The right side shows the difference in the ratio of the functional abundance in the 95% confidence interval. The functional group shown in red indicates *p* < 0.05.

**Fig 8:**
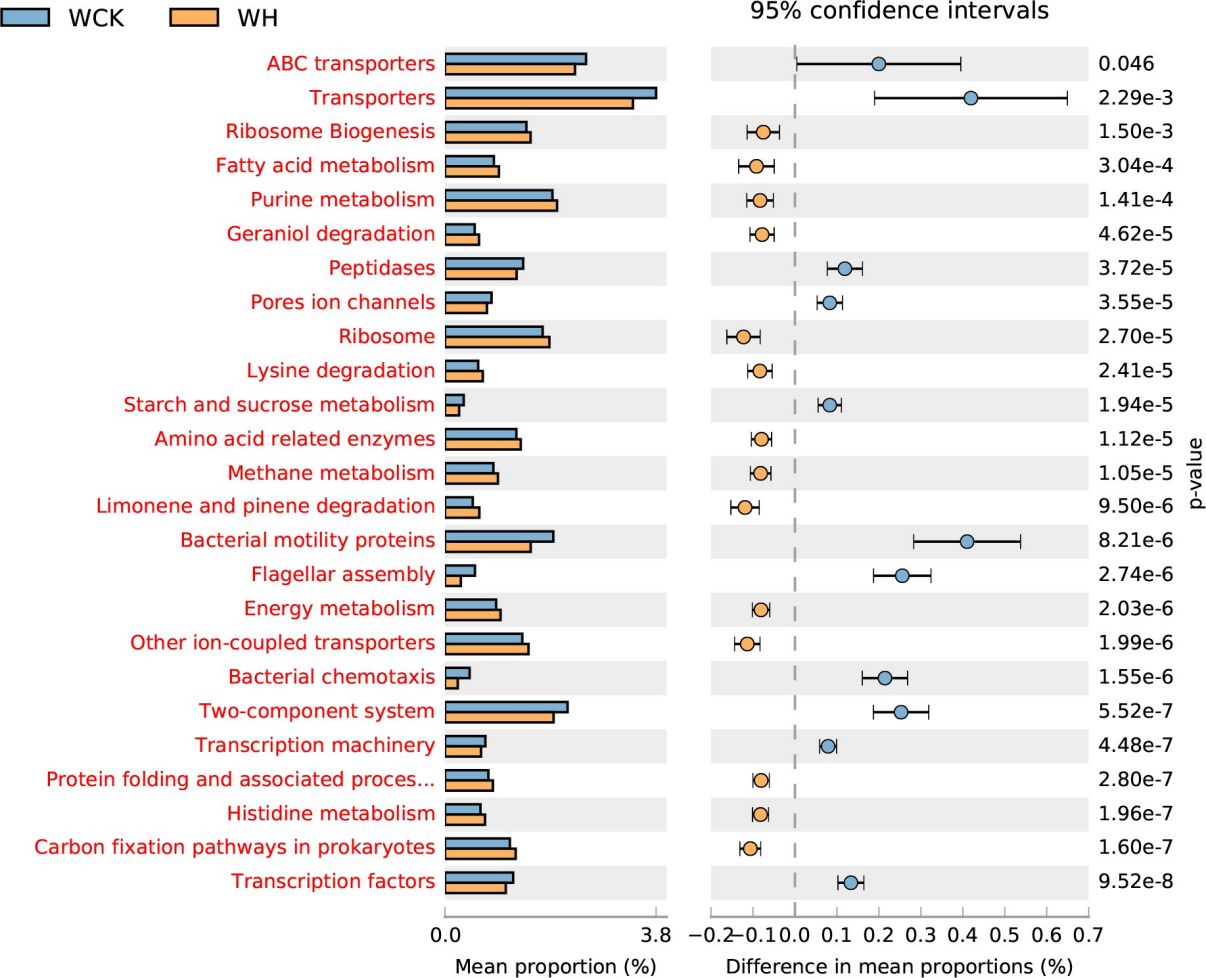
Top-25 functional abundance-difference analysis between the WCK and WH. WH denotes the antibiotic-untreated PWNs receiving the H-level of α-pinene. WCK denotes the antibiotic-untreated PWNs in the control group. The left side of the chart shows the abundance of different functional groups in the two groups of samples. The right side shows the difference in the ratio of the functional abundance in the 95% confidence interval. The functional group shown in red indicates *p* < 0.05.

**Fig 9:**
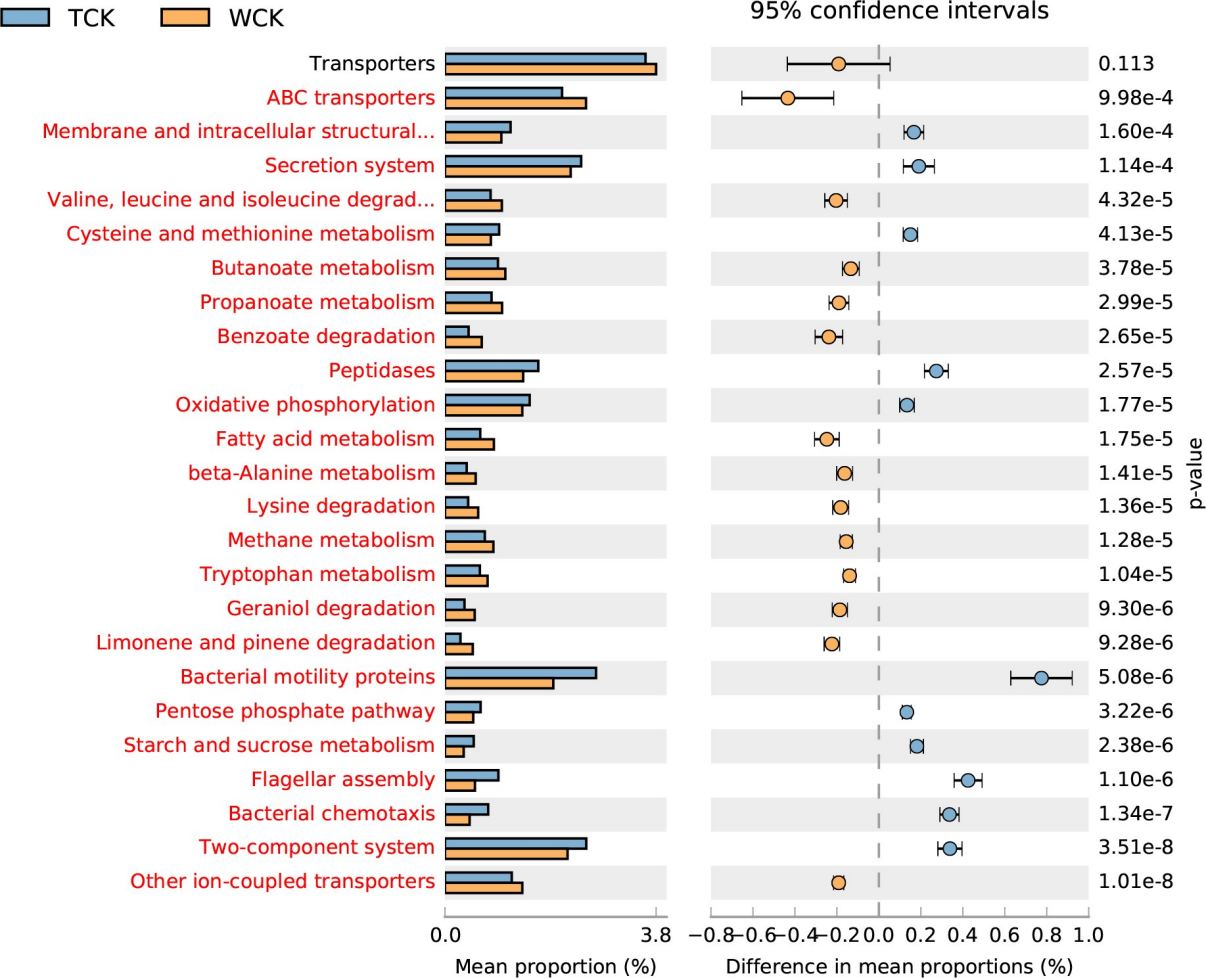
Top-25 functional abundance-difference analysis between TCK and WCK. TCK denotes the antibiotic-treated PWNs in the control group. WCK denotes the antibiotic-untreated PWNs in the control group. The left side of the chart shows the abundance of different functional groups in the two groups of samples. The right side shows the difference in the ratio of the functional abundance in the 95% confidence interval. The functional group shown in red indicates *p* < 0.05.

**Fig 10:**
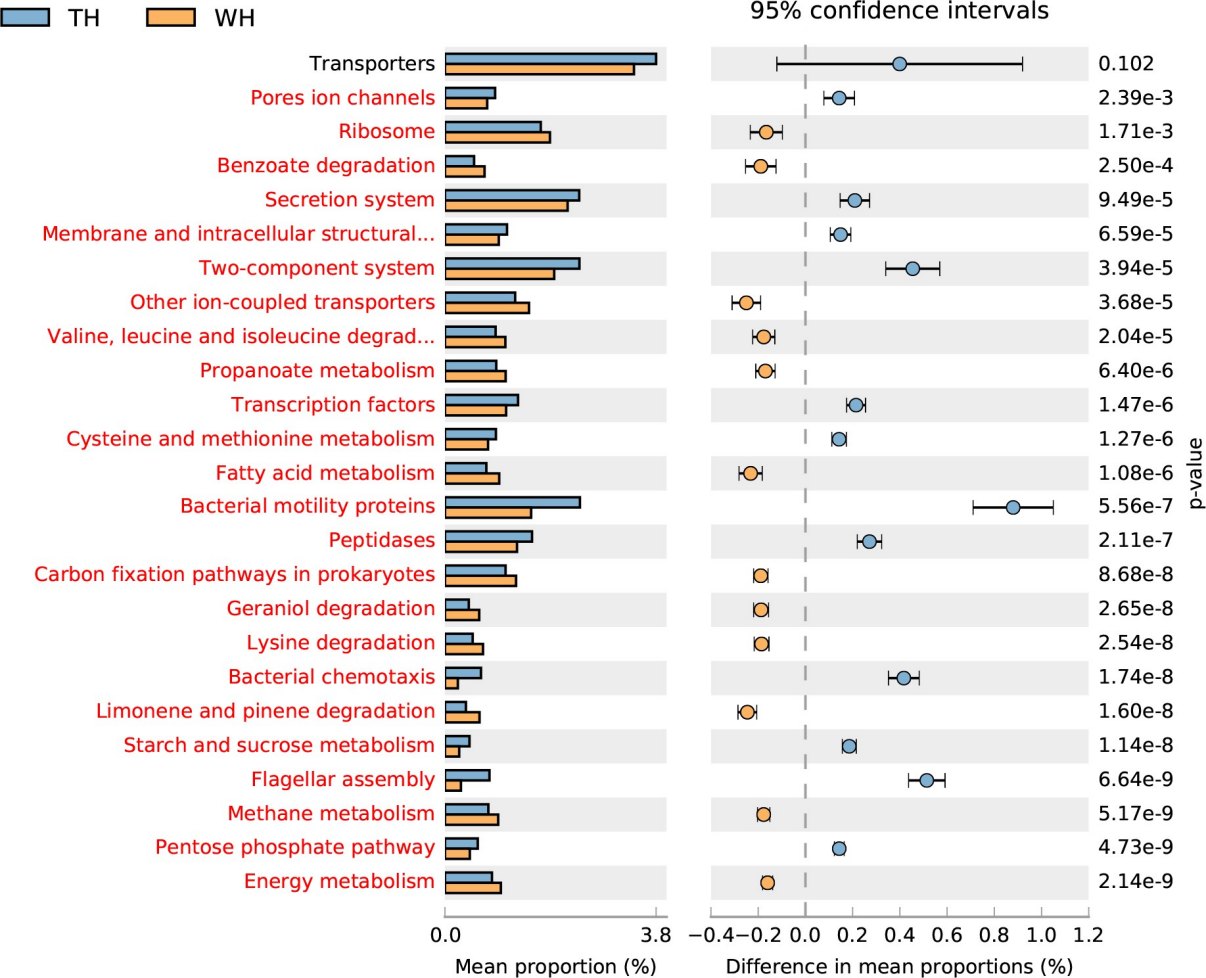
Top-25 functional abundance-difference analysis between TH and WH. TH denotes the antibiotic-treated PWNs receiving the H-level of α-pinene. WH denotes the antibiotic-untreated PWNs receiving the H-level of α-pinene. The left side of the chart shows the abundance of different functional groups in the two groups of samples. The right side shows the difference in the ratio of the functional abundance in the 95% confidence interval. The functional group shown in red indicates *p* < 0.05.

Differences in the functional abundance between the samples were compared based on the third-level KEGG pathway results of the PICRUSt function. The top-25 functional abundance-difference analysis revealed that the abundances of limonene and pinene degradation in the two groups treated with H-level α-pinene were significantly higher than those of their controls (Fig 7, Fig 8). Moreover, the difference between the control antibiotic-untreated PWNs and the H-level pinene antibiotic-untreated PWNs was larger than the difference between the control antibiotic-treated PWNs and the H-level pinene antibiotic-treated PWNs. The abundance of limonene- and pinene-degradation metabolism in the antibiotic-untreated PWNs was significantly higher than that of the antibiotic-treated PWNs, both in the control and H-level pinene comparisons (Fig 9, Fig 10).

## Discussion

The influences of α-pinene on PWN mortality rate and symbiotic bacterial communities were investigated in this study. The results showed that the mortality rate of PWNs increased with increasing concentrations of α-pinene. The antibiotic-untreated PWNs were more sensitive to α-pinene than that of the antibiotic-treated PWNs. For the antibiotic-untreated PWNs, the concentration of bacteria (CFUs) at the M-level of pinene reached the maximum among the experimental groups, but the community diversity was the lowest (Shannon and Simpson indexes). The results showed that there may be a few specific genera of bacteria associated with the antibiotic-untreated PWNs, which may increase significantly when treated with M-level pinene. Significant changes in the number of these bacteria may affect the mortality of nematodes.

The present study used fumigation for α-pinene treatments because of the volatile nature of α-pinene and the way in which PWNs are affected in trees. The results showed that α-pinene applied by fumigation had a certain degree of influence on the survival of nematodes, which differed slightly from previous results obtained by adding nematodes directly to α-pinene solution (LD_50_ > 20 mg mL^−1^) [11]. The nematocidal activity of pinene was low (LC_50_ > 1.0 mg mL^−1^) with treatment of 26 monoterpenoids, including pinene [23]. In the present study, α-pinene had low toxicity to PWNs over a short time period, which is consistent with previously reported results [11,23]. Pinene also has no obvious toxicity in *C*. *elegans* [24]. The toxicity of eight monoterpenes to *Callosobruchus maculatus* was tested by fumigation, and the results showed that pinene had the lowest toxicity (LD_50_ = 31.4 μL L^−1^) [25].

For the antibiotic-untreated PWNs, the concentration of bacteria for the M-level pinene was the highest, but the community diversity was the lowest. This result indicated that most bacteria were inhibited by pinene treatment. Nevertheless, there may be a few types of bacteria that are not sensitive to pinene treatment, and the number of bacteria can increase significantly at medium and low levels of pinene treatment. However, in nematodes treated with antibiotics, the activity of these bacteria was lost in the present study.

The importance of symbiotic bacteria in host fitness and adaptation has been widely acknowledged. The rapid development of high-throughput sequencing technology has been applied to investigating the symbiotic bacteria of PWNs [15, 26]. Bacterial species identified by high-throughput sequencing are more abundant than those identified by traditional culture methods [27–29]. Therefore, in the present study, high-throughput sequencing was used to analyze the community structure of the symbiotic bacteria in PWNs. The results showed that *Pseudomonas* spp. and *Stenotrophomonas* spp. were the dominant genera. The prevalence of these two genera is mostly consistent with the results of previous studies on dominant species of symbiotic bacteria of PWNs in China [26, 30, 31]. *Pseudomonas* sp. increased under treatment with pinene, while *Stenotrophomonas* spp. and *Delftia* spp. decreased in response to pinene treatment. According to the results of functional predictions, the abundance of limonene- and pinene-degradation metabolism in the antibiotic-untreated PWNs was significantly higher than that of the antibiotic-treated PWNs. Many previous studies have shown that *Pseudomonas* sp. has the ability to degrade α-pinene [32–36]. Therefore, *Pseudomonas* sp. in PWNs may also able to degrade α-pinene.

The symbiotic bacteria of PWNs are considerably abundant. The stable community structure is beneficial to the survival and development of the nematode. PWNs can stimulate the pine to produce terpenoid-secondary metabolites [4–8, 12]. A low concentration of compounds can inhibit bacterial invasion and provide time and space for nematode reproduction. A large number of terpenoids can affect the stability of symbiotic bacteria in nematodes, thus affecting the number of nematode populations. To our knowledge, this study is the first to have tested the toxicity of pinene to PWNs by fumigation and to have detected resultant changes of their symbiotic bacteria. This study provides data that will support further studies on the relationships among PWNs, symbiotic bacteria, and hosts.

## Conclusions

We conclude that α-pinene exhibits low toxicity to PWNs, and that *Pseudomonas* spp. and *Stenotrophomonas* spp. (as the dominant genera) were not sensitive to α-pinene. Future studies should explore the potential roles of dominant bacterial strains in PWNs, especially pinene-tolerant strains.

## Supporting information

S1 Fig Bacterial composition at the phylum level. WH, WM, WL, and WCK denote the antibiotic-untreated PWNs receiving the three different levels of α-pinene, as well as the control (WCK). TH, TM, TL, and TCK denote the antibiotic-treated PWNs receiving the three different levels of α-pinene, as well as the control (TCK).
(TIF)

S2 Fig Bacterial composition at the class level. WH, WM, WL, and WCK denote the antibiotic-untreated PWNs receiving the three different levels of α-pinene, as well as the control (WCK). TH, TM, TL, and TCK denote the antibiotic-treated PWNs receiving the three different levels of α-pinene, as well as the control (TCK).
(TIF)

S3 Fig Experimental flow diagram. (TIF)

S1 Table Data of nematode mortality and CFUs. (PDF)

S2 Table Analysis of the variance of nematode mortality rate. (PDF)

S3 Table Species abundance analysis of bacteria. (PDF)

S4 Table Analysis of the variance of OTUs. (PDF)
