# Supplementary figures and images for: Effects of α-pinene on the pinewood nematode (Bursaphelenchus xylophilus) and its symbiotic bacteria
Source: PLoS One. 2019 Aug 19;14(8):e0221099. doi: 10.1371/journal.pone.0221099 (PMC6699699; doi:10.1371/journal.pone.0221099)

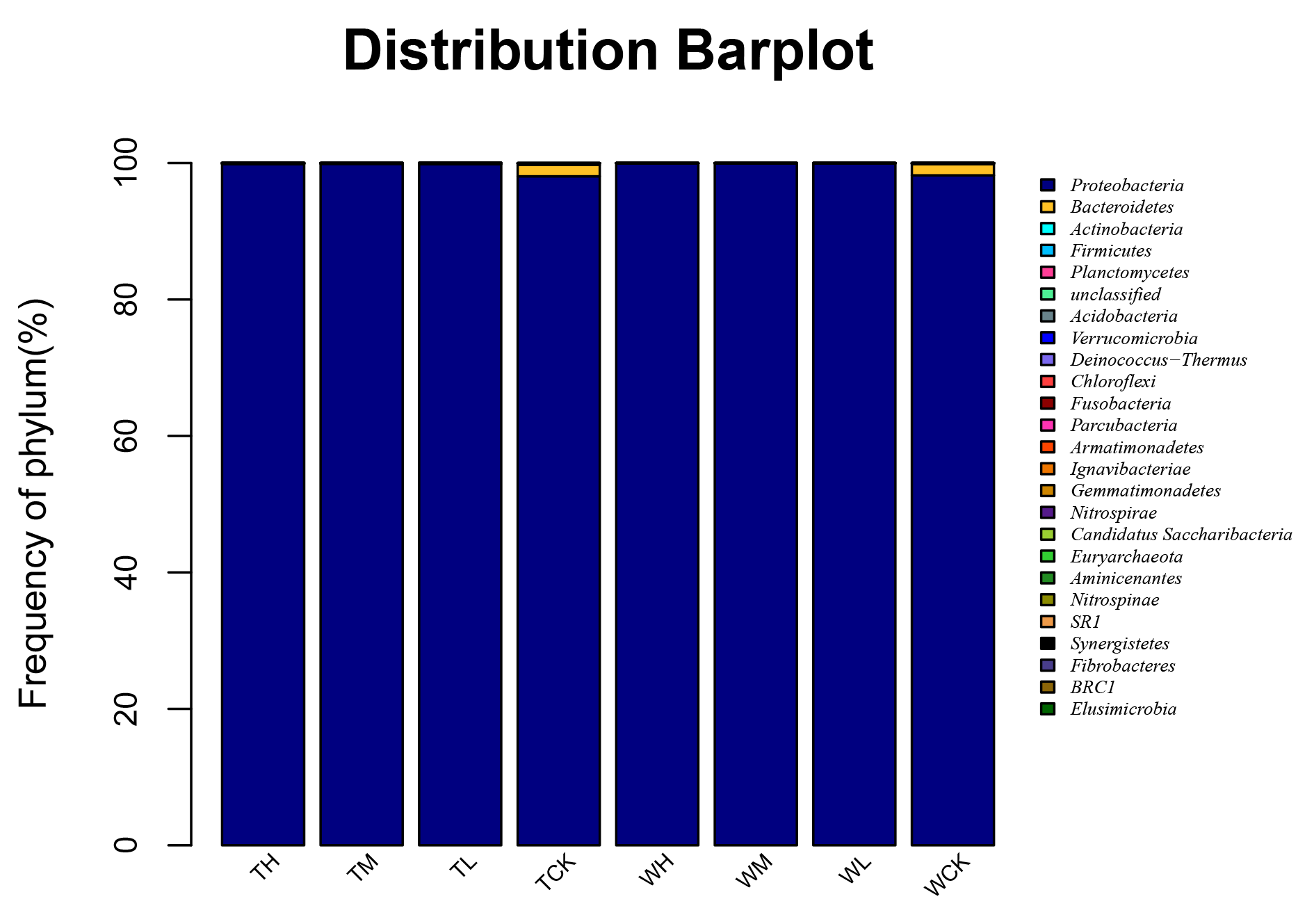

Supplement: S1 Fig — WH, WM, WL, and WCK denote the antibiotic-untreated PWNs receiving the three different levels of α-pinene, as well as the control (WCK). TH, TM, TL, and TCK denote the antibiotic-treated PWNs receiving the three different levels of α-pinene, as well as the control (TCK). (TIF) [file pone.0221099.s001.tif]

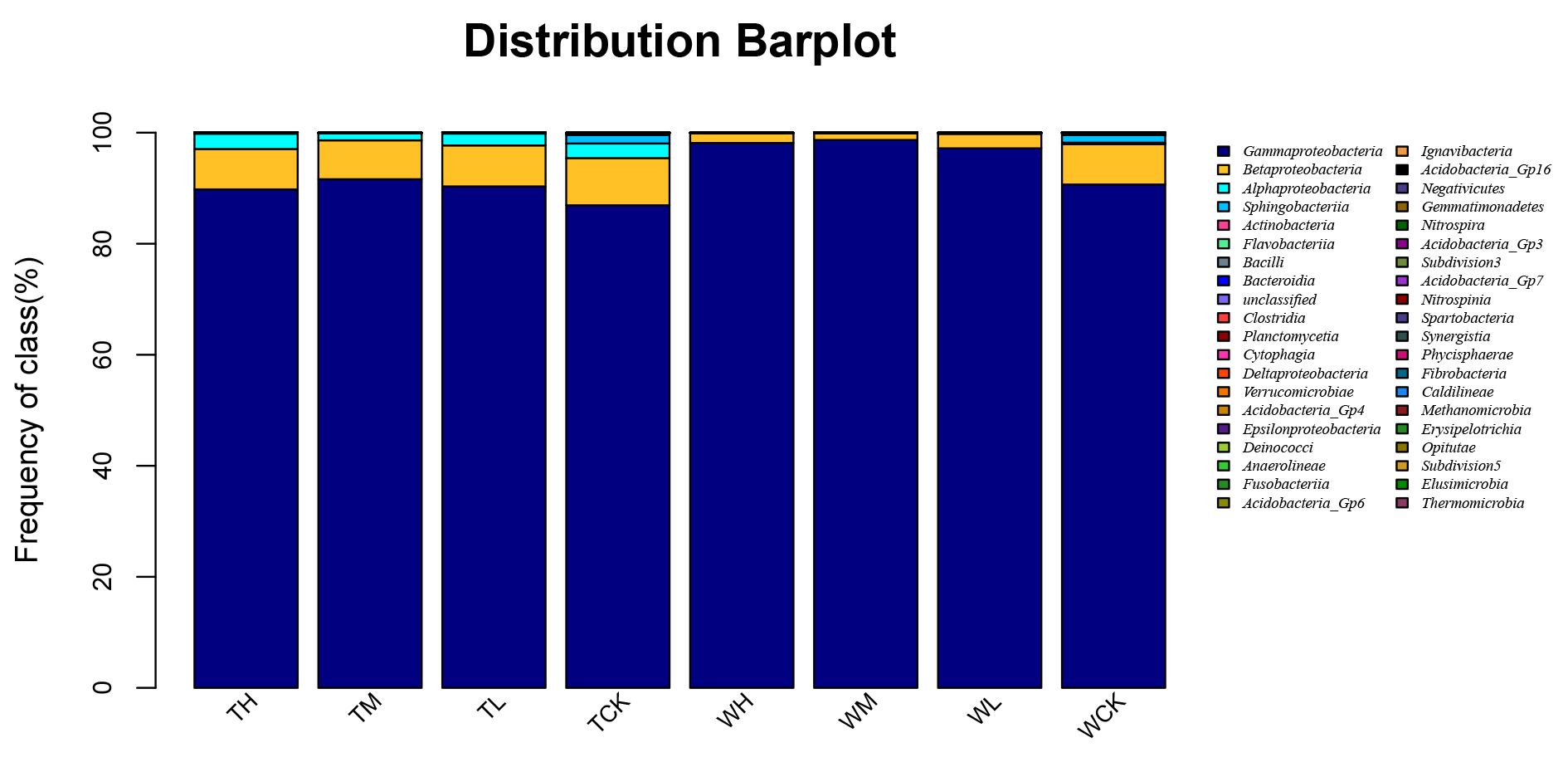

Supplement: S2 Fig — WH, WM, WL, and WCK denote the antibiotic-untreated PWNs receiving the three different levels of α-pinene, as well as the control (WCK). TH, TM, TL, and TCK denote the antibiotic-treated PWNs receiving the three different levels of α-pinene, as well as the control (TCK). (TIF) [file pone.0221099.s002.tif]

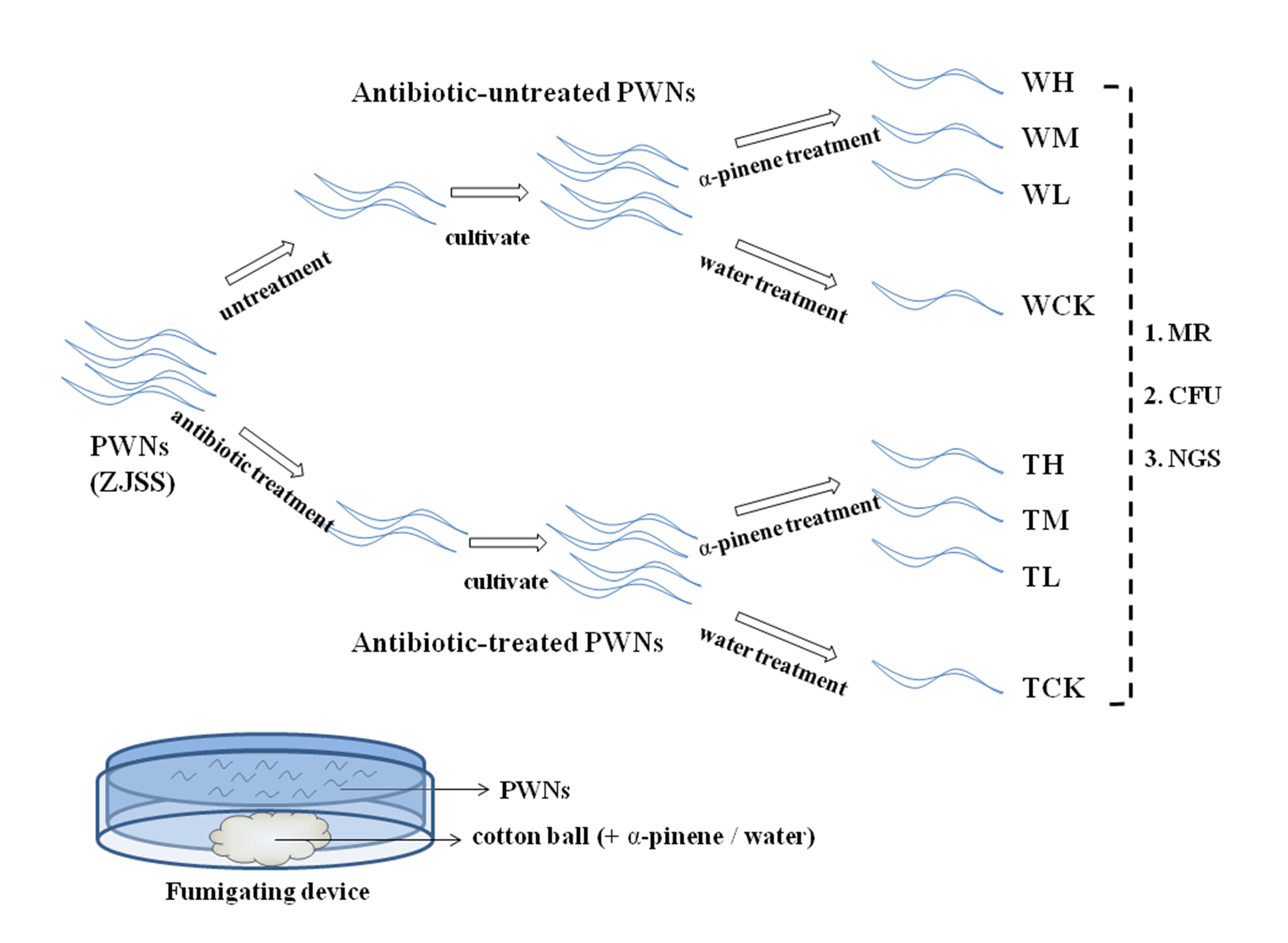

Supplement: S3 Fig — (TIF) [file pone.0221099.s003.tif]
