# Supplementary material for: Effects of α-pinene on the pinewood nematode (Bursaphelenchus xylophilus) and its symbiotic bacteria
Source: PLoS One. 2019 Aug 19;14(8):e0221099. doi: 10.1371/journal.pone.0221099 (PMC6699699; doi:10.1371/journal.pone.0221099)
Supplement: S1 Table — (PDF) [file pone.0221099.s004.pdf]

S1 Table. Data of nematodes mortality rate and CFU

PWNs group	Amount of α-pinene	Mortality rate of nematode	CFUs (per nematode)
Antibiotic-untreated PWNs	H-level	0.4910^{C} ± 0.057	422^{A} ± 30.502
	M-level	0.3903^{BC} ± 0.080	891^{B} ± 79.719
	L-level	0.3765^{B} ± 0.082	738^{AB} ± 216.477
	Control	0.2527^{A} ± 0.021	613^{AB} ± 188.432
Antibiotic-treated PWNs	H-level	0.6019^{b} ± 0.034	180^{a} ± 23.805
	M-level	0.6048^{b} ± 0.070	232^{b} ± 23.130
	L-level	0.5114^{a} ± 0.045	238^{ab} ± 57.546
	Control	0.4594^{a} ± 0.042	197^{ab} ± 26.662

Different letters indicate significant (p<0.05) differences in CFU among groups based on Tukey's HSD test.
