# Supplementary material for: Effects of α-pinene on the pinewood nematode (Bursaphelenchus xylophilus) and its symbiotic bacteria
Source: PLoS One. 2019 Aug 19;14(8):e0221099. doi: 10.1371/journal.pone.0221099 (PMC6699699; doi:10.1371/journal.pone.0221099)
Supplement: S2 Table — (PDF) [file pone.0221099.s005.pdf]

S2 Table. Analysis of the variance of nematodes mortality rate.

Tests of Between-Subjects Effects

Dependent Variable: Nematode mortality rate

Source	Type III Sum of Squares	df	Mean Square	F	Sig
Corrected Model	0.499[a]	7	0.071	21.756	0.000
Intercept	8.493	1	8.493	2592.732	0.000
Amount of pinene	0.200	3	0.067	20.310	0.000
PWNs group	0.280	1	0.280	85.339	0.000
Amount of pinene * PWNs group	0.020	3	0.007	2.007	**0.133**
Error	0.105	32	0.003		
Total	9.097	40			
Corrected Total	0.604	39			

a. R Squared = 0.826 (Adjusted R Squared = 0.788)
