# Supplementary material for: Effects of α-pinene on the pinewood nematode (Bursaphelenchus xylophilus) and its symbiotic bacteria
Source: PLoS One. 2019 Aug 19;14(8):e0221099. doi: 10.1371/journal.pone.0221099 (PMC6699699; doi:10.1371/journal.pone.0221099)
Supplement: S3 Table — (PDF) [file pone.0221099.s006.pdf]

S3 Table. Species abundance analysis of bacteria.

PWNs Group	Amount of pinene	Sample	Raw_num	Clean_num	Mean_len	Seq_num	Filtered_num	OTU_num
Antibiotic-treated PWNs	**H**	**TH6**	55,695	54,192	426.8	54,192	53,900	197
	**H**	**TH7**	52,869	51,609	428.35	51,609	50,421	158
	**H**	**TH8**	53,640	52,394	428.68	52,394	50,370	139
	**H**	**TH9**	56,531	55,031	428.58	55,031	54,523	194
	**H**	**TH10**	54,478	52,980	428.66	52,980	51,115	196
	**M**	**TM6**	55,358	53,883	428.69	53,883	53,179	189
	**M**	**TM7**	51,644	50,254	428.32	50,254	48,580	183
	**M**	**TM8**	52,218	50,972	428.52	50,972	49,014	172
	**M**	**TM9**	55,294	53,954	428.62	53,954	53,560	221
	**M**	**TM10**	52,069	50,557	428.44	50,557	48,954	146
	**L**	**TL6**	55,297	54,023	428.7	54,023	53,354	167
	**L**	**TL7**	56,879	55,391	428.48	55,391	53,961	181
	**L**	**TL8**	45,781	44,633	428.55	44,633	43,718	171
	**L**	**TL9**	51,646	50,315	428.07	50,315	50,087	197
	**L**	**TL10**	60,506	58,847	428.02	58,847	57,914	177
	**Control**	**TCK6**	52,034	50,692	428.57	50,692	50,563	145
	**Control**	**TCK7**	43,477	42,251	427.94	42,251	37,100	155
	**Control**	**TCK8**	55,976	54,568	428.19	54,568	54,437	138
	**Control**	**TCK9**	58,180	56,660	427.7	56,660	56,232	156
	**Control**	**TCK10**	54,400	52,846	427.52	52,846	50,758	150
Antibiotic-untreated PWNs	**H**	**WH1**	56,927	55,510	429.1	55,510	55,299	190
	**H**	**WH2**	58,079	56,728	428.97	56,728	56,425	260
	**H**	**WH3**	52,393	51,292	428.96	51,292	51,064	192
	**H**	**WH4**	56,775	55,405	428.98	55,405	54,259	228
	**H**	**WH5**	56,174	54,585	428.88	54,585	54,050	238
	**M**	**WM1**	52,240	51,079	429.11	51,079	50,242	263
	**M**	**WM2**	57,796	56,187	428.92	56,187	54,931	264

	**M**	**WM3**	54,014	52,746	428.85	52,746	52,225	220
	**M**	**WM4**	46,190	44,976	428.81	44,976	44,706	208
	**M**	**WM5**	61,246	58,512	428.91	58,512	58,303	267
	**L**	**WL1**	61,332	59,733	428.97	59,733	59,637	243
	**L**	**WL2**	55,043	53,737	428.89	53,737	53,001	259
	**L**	**WL3**	42,551	41,624	428.9	41,624	40,128	203
	**L**	**WL4**	49,250	47,671	428.86	47,671	46,315	242
	**L**	**WL5**	39,722	38,874	428.96	38,874	38,611	149
	**Control**	**WCK1**	56,567	55,241	428.71	55,241	54,399	215
	**Control**	**WCK2**	62,917	61,378	428.78	61,378	59,689	233
	**Control**	**WCK3**	57,941	56,738	428.87	56,738	55,956	203
	**Control**	**WCK4**	54,702	53,378	428.62	53,378	52,185	201
	**Control**	**WCK5**	52,253	50,467	428.67	50,467	49,203	254
**SUM**			2,158,084	2,101,913	17,143.12	2,101,913	2,062,368	7964
**AVERAGE**			53,952.1	52,547.825	428.578	52,547.825	51,559.2	199.1
