# Supplementary material for: Effects of α-pinene on the pinewood nematode (Bursaphelenchus xylophilus) and its symbiotic bacteria
Source: PLoS One. 2019 Aug 19;14(8):e0221099. doi: 10.1371/journal.pone.0221099 (PMC6699699; doi:10.1371/journal.pone.0221099)
Supplement: S4 Table — (PDF) [file pone.0221099.s007.pdf]

S4 Table. Analysis of the variance of OTUs.

**Tests of Between-Subjects Effects**

Dependent Variable: OTUs

| Source | Type III Sum of Squares | df | Mean Square | F | Sig |
|---|---|---|---|---|---|
| Corrected Model | 36331.975[a] | 7 | 5190.282 | 7.234 | .000 |
| Intercept | 1589218.225 | 1 | 1589218.225 | 2215.054 | .000 |
| **PWN groups** | **30747.025** | **1** | **30747.025** | **42.855** | **.000** |
| Amount of pinene | 3757.275 | 3 | 1252.425 | 1.746 | .177 |
| PWN groups * Amount of pinene | 1827.675 | 3 | 609.225 | .849 | .477 |
| Error | 22958.800 | 32 | 717.462 | | |
| Total | 1648509.000 | 40 | | | |
| Corrected Total | 59290.775 | 39 | | | |

a. R Squared = .613 ( Adjusted R Squared = .528 )
